# The Moderating Effects of Students’ Personality Traits on Pro-Environmental Behavioral Intentions in Response to Climate Change

**DOI:** 10.3390/ijerph14121472

**Published:** 2017-11-29

**Authors:** Tai-Yi Yu, Tai-Kuei Yu

**Affiliations:** 1 Department of Risk Management and Insurance, Ming Chuan University, 250 Zhong Shan N. Rd., Sec. 5, Taipei 111, Taiwan; yti@mail.mcu.edu.tw or yutaiyi@gmail.com; 2 Department of Business Administration, National Quemoy University, One University Road, Jinning Township, Kinmen Hsien 892, Taiwan

**Keywords:** climate change, personality traits, moderator effect, pro-environment behavioral intentions, sustainability value

## Abstract

This study developed a model that examined the relationship between undergraduate students’ beliefs, norms and pro-environment behavioral intentions in the context of global climate change (GCC). The model was further evaluated to determine whether latent variables, such as sustainability value, environmental concern, social norms, perceived risk, pro-environmental attitude, as defined by the theory of planned behavior and value-belief-norm theory, significantly influenced students’ intentions towards pro-environmental behavior. The research model was empirically tested using data collected form 275 undergraduate students. Empirical results found support for four interaction effects of personality traits and the related latent variables of environmental attitude, including sustainability value, social norms, environmental concern and perceived risk. The impact of undergraduate students’ environmental attitudes was moderated by personality traits. The findings of this research offer policy makers and enterprises better understandings of undergraduate students’ attitudes and behavioral intentions towards GCC and promote the visibility of this issue.

## 1. Introduction

Global climate change (GCC), a public risk that affects the entire world, is a phenomenon that is mostly attributable to human activity that has occurred over the past century [1]. GCC strongly affects multiple factors that influence human and other organisms’ health and survival, including energy resources, space, water, air, food, sanitation and the natural environment [2]. People worldwide have been voicing concerns about global warming and a growing number of them have shown a willingness to act on their concerns [3,4,5,6,7]. Educators concerned about sustainability hope to understand whether undergraduate students perceive GCC as a risk and what behavioral changes these students are willing in the face of GCC. How people learn to think about sustainability guides their behavioral intentions and behaviors as local and global citizens. For instance, Wright and Wilton [8] conducted in-depth interviews with 37 facility management directors of Canadian universities and found that almost all participants felt that environmental sustainability was a vital part of sustainable development and often mentioned resource use and waste reduction. Barr [9] found that reduction and reuse pro-environmental behaviors were predicted by the individual’s underlying environmental values, knowledge and concern-based variables. Recycling pro-environmental behavior was, in contrast, characterized as a highly normative behavior. Horhota et al. [10] indicated that communication/awareness, inconvenience, financial concerns and lack of engagement are the four key behavioral barriers to sustainable actions. Wachholz et al. [11] demonstrated that most college students express environmental concern about climate change; however, students still hold common misconceptions about the basic causes and consequences of climate change.

Education about sustainability aims to shape students using the knowledge, attitudes, commitments, motivations, technologies and strategies that are necessary to solve environmental problems [10,11,12]. Studies have shown that sustainability education can shape attitudes towards sustainable consumption and raise the need for consumption-related lifestyle changes for undergraduate and high school students [13]. Thus, this current research was motivated by the following questions:

Research question 1: What environmental beliefs about climate change cause pro-environmental behavior intentions and even taking actions meant to GCC adaptations?
Research question 2: How do personality traits moderate the influence of environmental beliefs on environmental attitude?

The establishment of a pro-environmental behavior model could promote GCC adaptation strategies and change opinions about green products and sustainable development. A pro-environmental behavior model can promote a better understanding of the relations among sustainability values, environmental concerns, environmental attitude and behavioral intentions. Specifically, this study introduces a new conceptual framework useful for examining pro-environment behavioral intentions of undergraduate students, which is based on understanding people’s beliefs about climate change issues and their attitudes toward making adaptation actions. This model can improve initiatives, sustainable development and environmental education, all of which are necessary for effective action against climate change.

## 2. Literature Review

### 2.1. Theories of Pro-Environment Behavioral Intentions

An earlier model of environmental behavior [14], which considered three factors of knowledge, awareness (or attitude) and behavior, proposed that increasing an individual’s knowledge about environment leads to a change in attitudes, increases the level of awareness and changes the person’s behaviors. Ajzen’s [15] theory of planned behavior (TPB) explains behavioral intentions using three factors: attitude, subjective norms and perceived behavioral control; this theory suggests that an individual’s prior attitudes towards GCC influence how much the person attempts to change related attitudes and beliefs [4,16,17]. One demonstrable method of influencing pro-environmental behaviors is a norms-based intervention. Norms may influence the behavioral intention of undergraduate students, particularly with regard to highly valued behaviors. Thus, according to the TPB, individuals holding positive attitudes toward pro-environmental activism have normative support for pro-environment behavioral intentions. Shove [18] asserted that the ABC paradigm (attitude, behavior and choice) within the TPB may be useful in explaining the role of attitudes and other relationships of GCC-related behaviors of students. Shove [18] also examined the extent to which environmental beliefs can be used to help encourage support for action against climate change. Ajzen [15,19] demonstrated that behaviors are manifestations of the values and final attitudes of individuals. Mancha and Yoder [20] reported on antecedent beliefs and their related behavioral intentions.

Stern [21] proposed the value-belief-norm theory (VBN), which is another theoretical framework applicable to pro-environmental behavior. The VBN theory includes norm activation theory [22] and is related to strength of self-enhancement values—this theory uses more complex causal relationships than the TPB model to explain pro-environment behavioral intentions. These latent variables include ethics, values, beliefs and norms that favor environmental protection [23,24]. Steg et al. [25] used the VBN theory to evaluate families’ level of acceptance of energy policies targeting carbon reduction and confirmed that all VBN variables (environmental value, awareness and ascription of responsibility to self-beliefs) affected behaviors, beliefs, norms and adoption. Steg et al. [24] claimed that the strength of normative goals depends on environmental values and situational factors (e.g., biospheric values, situational cues, behavioral costs) that influence the accessibility of sustainability value. As a result, the VBN theory is currently the most widespread model used to investigate the determinants of environmental behavior. Furthermore, the relationships between the latent variables of VBN theory illustrate the effect that environmental values have on individual evaluations of the consequences of environmental behavior.

Taylor and Todd [26] found that a more favorable attitude toward an environmental action results in a perception of high relative advantage and low complexity and sustainability values and environmental concerns significantly affect willingness to adopt pro-environment attitude. Sustainability value and environmental concern must provide benefits that supersede those of inconvenience and may include economic benefits and personal motives [27]. An individual’s value priorities might be determined by observing their direct attention to consequences, the sustainability value structure that underlies pro-environment behavioral intentions may guide pro-environmental beliefs, norms and attitudes, which in turn affect behavioral intention. Individuals are likely to focus on the environmental consequences of alternative strategies and to evaluate these consequences as dominant issues in order to determine pro-environmental actions. Steg et al. [24] found that individuals with strong environmental values are relatively more motivated to adopt pro-environmental attitude. Likewise, environmental values seem to affect pro-environmental attitude due to information or awareness related to the consequences of pro-environmental actions. These attitudes involve verbally encouraging/motivating one or more persons to take a desired pro-environmental action [20].

Most studies [28,29,30] of pro-environmental behavior that use VBN theory have found a positive relationship between the degree of environmental concern and willingness to enact pro-environment attitude. Inherent to the idea of environmental concern is the recognition of people in relation to nature and of the reciprocal threats of environmental deterioration and ecological catastrophe. People who are concerned both for the welfare of others and for biospheric values tend to engage in pro-environmental attitudes because they believe that protecting the environment is the right thing to do [24,31,32]. Empirical evidence validates the claim that environmental value and environmental concern have significant influences on explaining specific beliefs and behaviors and may, therefore, be predictors for various variables, such as pro-environmental attitude. Sustainability values could be depicted using three core values-economics, society and environment [33]. The economic value pertained to materialism [34], the environmental value pertained to environmentalism and the social value pertained to equity [35]. The equity value stated to how actions of adaptation strategies affect social welfare and surrounding communities [35]. As respondents in this study are undergraduate students and therefore they lack a clear and deep understanding of equity, this latent variable was not measured in this study. Sustainability value in this study was hence composed of environmentalism and materialism. Therefore, the following hypotheses were made:

**Hypothesis** **1.** *An individual’s sustainability value is positively related to pro-environmental attitude.*

**Hypothesis** **2.** *An individual’s social norms are positively related to their pro-environmental attitude.*

**Hypothesis** **3.** *An individual’s environmental concerns are positively related to their pro-environmental attitude.*

The perceived risk of an environmental problem may affect an individual’s willingness to adopt behaviors that are more environmentally friendly. Environmental scientists have found that individuals respond to hazards based on perceived risk and that there exists a significant gap between the public’s perceived risk and their pro-environment behavioral intentions on the issue of GCC [4,36,37,38,39]. This gap is known as the Giddens paradox [40] because the adverse effects of GCC are not immediately evident and it describes the disconnection that exists between the perceived risks of GCC and cognitive style [41,42]. Previous research on perceived risk has found only a weak relationship between environmental attitude and perceived risk across a variety of environmental issues [6,37]. Indeed, the public may not grasp the full implications of GCC immediately because the effects of GCC do not immediately manifest and the fact that long-term monitoring of these effects and their impacts on human behavioral intention and activities are required [38,43]. The knowledge deficit model found that an individual’s perceived risk of GCC tends to be excessive when they lack behavioral knowledge [11,43,44,45]. Stedman [7] used a linear regression model to explain how the policy making process in Canada copes with climate change and the perceived risks of GCC. Giddens [40] and Hutchinson et al. [46] argued that cognitive perception, global public goods and temporal discounting are the main factors influencing the public’s perceived risks of GCC and their pro-environment behavioral intentions. These literatures show that perceived risk is a critical factor affecting pro-environment behavioral intentions. Based on this discussion, the following hypothesis was formed:

**Hypothesis** **4.** *An individual’s perceived risk of GCC is positively related to environmental attitude.*

López-Mosquera and Sánchez [47] used the TPB and VBN theories to study the willingness of park visitors to contribute to the costs of park conservation. The results suggest that positive attitudes, strong altruistic values, strong environmental beliefs and social norms are critical factors influencing visitors’ willingness. Moreover, researchers have found that values affect the strength of goals within the context of a particular environmental issue as well as perceived risk [24,32,37]. Kaiser et al. [48] used TPB and VBN to explain conservation behaviors and to highlight the differences between the two models. In the TPB, behavioral intention accounted for 95% of conservation behaviors, while in VBN, norms accounted for 64% of conservation behaviors. However, TPB better described the correlations among the variables than VBN. Drawing from the original formulation of the TPB, several studies have supported the precedence of environmental attitudes over environmental behavioral intention [17,20]. Based on the previous findings, the following hypothesis was formulated:

**Hypothesis** **5.** *An individual’s environmental attitudes are positively related to behavioral intentions.*

### 2.2. Moderating Effects of Personality Traits on Behavioral Intentions

Researchers have addressed the variables of pro-environmental behavior in a variety of ways, including focusing on the use of attitudes to induce participatory behaviors [28,30], examining the impact of pro-environment behavioral intentions on students’ background variables (gender, age and experience [12]) and studying littering attitudes as a potential moderating variable between personality traits (altruism and locus of control) and pro-environment behavioral intentions [30,49,50]. Additionally, Simmons and Widmar [51] used people’s recycling behaviors and holistic personality traits (e.g., conscientiousness) as main test variables. While participants were highly motivated when situational manipulations of environmental behaviors led to positive feedback, they did not continue practicing these behaviors in everyday life [43,52,53].

Personality traits explain some differences in individual actions under similar situations and can be used as predictors of individual behavior. The Big Five Inventory of personality [54] comprises the dimensions of neuroticism (in the current study this was called emotional stability), agreeableness, conscientiousness, openness to experience and extraversion, it seems personality traits are a multi-dimensional construct [54,55]. In addition, the second-order construct (personality traits) is a linear combination of five first order factors and the first-order factors themselves have reflective indicators; when consequences of the latent construct are included, the formative model can be estimated. Wood [56] argued that sustainable behavior may not immediately affect or stop GCC and that a person’s irresponsible behaviors are mainly due to personality traits. Milfont and Sibley [57] found that agreeableness and conscientiousness influenced self-reported energy-saving behavior and environmental commitment. Hirsh [58] indicated that higher environmental concern is significantly associated with higher levels of agreeableness, openness, stability and conscientiousness, with no significant association observed for extraversion. An individual’s personality traits may be a positive catalyst that enhances the effect perceived risk of GCC on pro-environmental behavior. These previous findings lend support to the assertion that the Big Five personality traits moderate the antecedent variables that influence pro-environmental attitude. Therefore, drawing from these previous findings, the following predictions were made:

**Hypothesis** **6.** *The relationship between sustainability value and pro-environmental attitude is moderated by level of personality traits. That is, the relationship is weaker with lower levels of personality traits and stronger with higher levels of personality traits.*

**Hypothesis** **7.** *The relationship between social norm and pro-environmental attitude is moderated by level of personality traits.*

**Hypothesis** **8.** *The relationship between environmental concern and pro-environmental attitude is moderated by level of personality traits.*

**Hypothesis** **9.** *The relationship between perceived risk and pro-environmental attitude is moderated by level of personality traits.*

**Figure 1:**
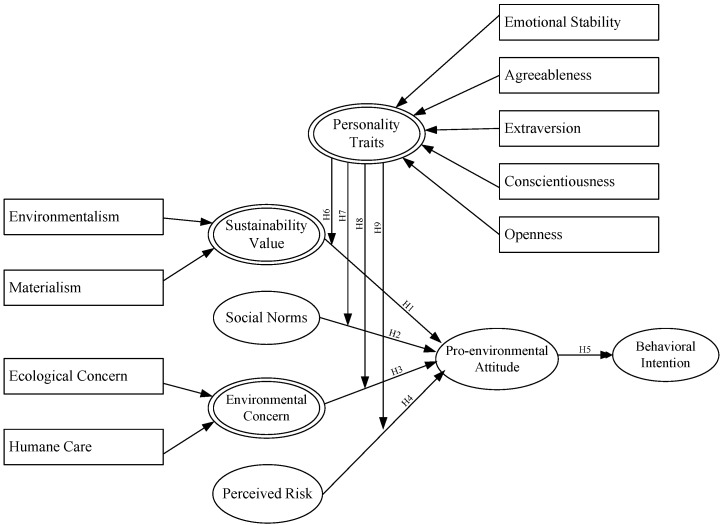
Conceptual of research model. H: Hypothesis.

In sum, the present study serves as a theoretical basis for predicting the pro-environment behavioral intentions of undergraduate students by realizing the interactions of latent variables and the moderating effects of personality traits on pro-environmental attitude (See Figure 1).

## 3. Methods

### 3.1. Analysis

Partial least squares (PLS) is a statistical method that is used to study or to construct linear models. PLS is able to simultaneously process sets of endogenous and exogenous variables. This study utilized SmartPLS2.0 software (SmartPLS GmbH, Bönningstedt, Germany) developed by Ringle et al. [59] to analyze both the measurement and structural models. PLS is recognized as an effective analytical technique and is most commonly associated with studies that focus on prediction as an outcome. Academic researchers increasingly use PLS path modeling because of its ability to model latent constructs under conditions of non-normality distribution with small to medium sample sizes [60]. PLS has minimal demands on the sample distribution requirements and sample size limitations imposed by other SEM techniques. Petter et al. [61] indicated that PLS analysis is based on the components-based model, whereas LISREL and AMOS models are based on the covariance-based model. The present study used structural equation modeling (SEM) implemented within PLS for data analysis, in order to obtain standard errors and *t*-values for the parameter estimates. A total of 275 valid samples were used. The approach defined by Cohen et al. [62], which draws on the three-step procedure of Marsh et al. [63], was used to examine the moderation effect. To ensure that multicollinearity between predictors and personality traits did not affect the statistic results, each variable was first mean-centered and interaction terms were based on centered scores.

### 3.2. Instrument Tools

After determining the theme of the research, a literature review was performed on the following topics: environmental values, attitudes and behavior chains; the perceived risks of GCC and personality traits. The questionnaire comprised previously published multi-item scales with favorable psychometric properties. This study used 10 personality inventory questions to measure the Big Five dimensions of personality. The constructs of environmental concern, sustainability value, environmental attitude and behavioral intention were measured using scales adapted from Ajzen [19], Steg et al. [24,25], Dunlap et al. [28], van Riper and Kyle [30], Liobikienė and Juknys [32], Gifford [64]. The scales for perceived risk and social norms were also adapted from prior research [11,19,24,38,64]. All of the survey questionnaire items were measured using 7-point Likert scales ranging from “strongly disagree” (1) to “strongly agree” (7). Next, a conceptual framework for the study was established. Three scholars examined the research concepts and questions and the instruments were revised according to their feedback. The face and content validity of the instrument was verified based on in-depth interviews with these professionals.

A questionnaire pretest was conducted to assess the content of the proposed study questionnaire (see Appendix A). The pretest goals included preventing participants from giving false answers or altering their answers due to semantic problems with the questionnaire. Undergraduate students who had taken a course in environmental education during the previous semester took the pretest. This course included at least 18 classroom hours about GCC issues. The pretest was conducted using face-to-face interviews and the participants were allowed to ask questions about the questionnaire at any point, to ensure that the participants had grasped the intentions properly and understood the semantics of the questions. In the first stage, participants answered the questions without any prompts given, although they were permitted to seek clarification on points that they found confusing. The second stage began after the participants had completed the questionnaire. Face-to-face interviews were carried out with 84 participants who were currently students at business schools in southern Taiwan. A total of 72 valid questionnaires remained after the deletion of incomplete questionnaires.

To ensure appropriate item validity and reliability, three of the items were removed and all of the items were randomly arranged in order to reduce the potential ceiling (or floor) effect, which induces monotonous responses in response to items that are designed to measure the same construct. Three measurement indicators were used to examine the questionnaire items. First, the correlation matrix for all questions was calculated. Any pair of questions with a relatively high degree of correlation (correlation coefficient greater than 0.9) was either combined into a single new question or one of the two questions was eliminated. Next, the top and bottom 25% of the scores comprised the high- and low-scoring groups, respectively. The difference between the mean values of the two groups was defined as the degree of discrimination and measurement variables with a low degree of discrimination were deleted. Finally, we deleted any questions with a commonality below 0.5, as suggested by Hair et al. [65]. We also followed these steps in the actual survey process in order to ensure the reliability and validity of the questions.

### 3.3. Participant Profiles

The participants in the study were all undergraduates currently studying at comprehensive universities and universities of science and technology in Taiwan. We excluded students who had not taken at least one course in environmental education. Qualified participants were selected before issuing the questionnaires, a total of 330 questionnaires were issued. Each questionnaire included 10 reverse questions and completed questionnaires were invalidated if more than 50% of the reversed questions were wrong. Further, completed questionnaires were invalidated if the respondent provided the same answers for over 90% of the questions. After filtering, a total of 275 valid surveys retained and used in the analysis, giving validity rate of 83.3% (see Table 1). All subjects gave their informed consent for inclusion before they participated in the study. All of participants in the study were completely voluntary; they receive the opportunity to refuse participation at any time without consequences. The study ethics procedures were carried out in accordance with the 1964 Helsinki declaration and its later amendments or comparable ethical standards. This questionnaire study, no ethical external approval was required under Taiwan’s law. This exemption was because the data was anonymous and there is no way for readers to be able to identify the participants. There are no name lists that correspond to the respondents of questionnaire and the names of the participant universities were not mentioned. All subjects were informed about the research and all participants include in the study provided informed consent.

## 4. Results

### 4.1. Reliability of the Research Model

Anderson and Gerbing [66] suggested that the following two points should be confirmed when analyzing measurement models: (i) the accuracy of the model in measuring latent variables and (ii) the convergent and discriminate validities for the test model. Environmental concerns and personality traits were formative indicators for the proposed model. The reliability indicator of the formative latent variables in this model could not be calculated. To measure the convergent and discriminant validity of the subscale, the other variables were calculated based on the recommendations of Bagozzi and Yi [67]. The three most common indicators (e.g., individual factor loading, composite reliability, average variance extracted) were used to evaluate the reliability and validity of the proposed model (see Table 2).

Along with the formative indicators, the loading capacities of all of the individual test variables exceeded the recommended value of 0.5; therefore, they were considered significant. The coefficients of the loading capacities of the sample factors ranged from 0.696 to 0.956, which corresponds with the value recommended by Hair et al. [65]. Higher composite reliability (CR) values are associated with higher internal consistency among latent variables. The CR values in the present study ranged from 0.783 to 0.981, which exceed the value of 0.6 recommended by Fornell and Larcker [68], indicating strong internal consistency within the model.

Using PLS for analysis, CR and AVE became the reliability and validity indicators for the measurement and structural models. The higher the average variance extracted (AVE), the greater the discriminant and convergent validities are among the latent variables. Fornell and Larcker [68] recommended an AVE above 0.5 and the AVE values of latent variables ranged from 0.510 to 0.910. Although the AVE values of interaction factors did not satisfy the strict convergent validity value (from 0.452 to 0.779), the composite reliability of interaction factors ranged from 0.905 to 0.960, indicating clear discriminant validity among the latent variables of the proposed model. The CR values of all variables in the present study exceeded 0.7, the benchmark AVE, implying that the results of the measurement model exhibited both convergent and discriminant validities.

The goodness-of-fit (GoF) indicator established by Tenenhaus et al. [69] was used to study the overall fit of the structural models with the analyzed variables. GoF is based on the minimum partial correlation method and uses maximum likelihood estimation to estimate the parameters. Marcoulides et al. [70] combined the AVE of Fornell and Larcker [68] with the benefits of the GoF indicator to first make improvements to and then to propose new standards for the GoF (poor = 0.1, average = 0.27, good = 0.42). The GoF of the present study was 0.568, which suggests an overall good fit for the model. Furthermore, due to the selection of values related to sustainability value and environmental concern as formative indicators, the structural model had an explanatory power of 1.00, with coefficients of the variables ranging from 0.387 to 0.521. Four of the five main hypothesized paths achieved significance. Considering the effects and interactions of the moderator variables, eight path relations reached significance.

Researchers use either multi-group analysis or the model invariance test to estimate the external validity of a proposed model and to test its stability. The present study used data from 275 valid samples. After the blindfolding function of the SmartPLS analysis tool set the same numbers for the omission distance and research variables, the cross-validated redundancy coefficient (*Q^2^*) of the dependent variables ranged from 0.231 to 0.910 (average: 0.646) and *Q^2^* ranged from 0.491 to 0.910 (average: 0.731). These values met the benchmark value requirements of *Q^2^* predictive relevance outlined by Chin et al. [71], indicating that the proposed model had adequate robustness and stability.

### 4.2. Testing the Moderator Effect of the Variables

Baron and Kenny [72] performed the test of the moderator effects of variables and suggested that standardized questions are required when applying multiple regression analyses and when using variables to test interaction effects. Kenny [73] suggested testing the level of significance of the independent variables, moderating variables and interaction effects separately when validating independent variables. Hence, three models were assigned for the transition to the moderating effects of the structural model. The first model included the independent variables and non-effects of interaction (step 1). The second model included the direct effects of the moderator variable (step 2). And the third model included both the moderator variable and the interaction effects (step 3). The present study assessed the interaction effects of the variables based on the recommendations of Chin et al. [71]. Although questionnaires were used, the characteristics of the structural equation models were considered in establishing the three models based on the aforementioned tri-level concept. Thus, the present study adopted a standardized path coefficient that was based on the proposed model.

Model 1: The proposed model specified all five causal paths, with three paths identified as statistically significant. Neither sustainability value nor environmental concern was found to have a statistically significant impact the pro-environmental attitude path.
Model 2: The significance of the aforementioned pathways did not change. However, the main effect of the newly added moderator variable (personality traits to pro-environmental attitude path) achieved significance.
Model 3: The effects of the interactions between personality traits and the four latent variables (sustainability value, social norms, environmental concern and perceived risk) and pro-environmental attitude all achieved statistical significance (see Table 3), demonstrating interaction effects for personality traits, sustainability value, social norms, environmental concern and perceived risk.

In examining the main effect of the moderator and independent variables, personality traits were found to have statistically insignificant impacts on the main effect of pro-environmental attitude. The relationship of sustainability value on pro-environmental attitude turned from negative to positive and this relationship remained insignificant. Personality traits moderated social norms on pro-environmental attitude path relationship, leading to expansion. The environmental concern on pro-environmental attitude path relationship turned from positive to negative due to the interaction effects with personality traits, leading to reversed expansion.

### 4.3. Path Relationships of the Proposed Model

The present study proposed and tested a hybrid and reflective indicator model that used formative indicators including environmental concern, sustainability value and personality traits. The concept of sustainability value covers the impact of human activities on the environment and individual beliefs about the natural ecology, which were categorized as two extremes of pure environmentalism and pure materialism, with greater/lesser degrees positioned between these poles. Sustainability values were linearly constructed with environmentalism (0.310) and materialism (0.777). Most undergraduate students believe that materialism has precedence over environmentalism in terms of their sustainability value and give prior consideration to the economic benefits of human activities and empirical data strongly supports this path relationship. In environmental behavior model, environmental concern encompasses human feelings toward the environment, ecology and other human beings. Ecological concern (0.852) and humane care (0.379) constituted environmental concern. As for the GCC issue of specific environmental beliefs, the present study found that high ecological awareness of general consequences of environmental concern and ecological concern was the primary guide for undergraduate students’ environmental concern. This result is consistent with de Groot and Steg [74], who respectively identified ecological concern as a good predictive variable for green behaviors and altruistic ecological activities.

The present study demonstrated no significant relationship between sustainability value and pro-environmental attitude, as participants gave very low scores for sustainability values (2.68 out of 7.0). In the context of GCC, the participants earned very high scores (5.57) for pro-environmental attitude, indicating a negative correlation between the two. Thus, the values that students hold regarding sustainability and environmentalism may be correlative. However, significant differences existed between materialism and environmentalism. The present study subsequently set these two different values on sustainability value into a linear combination and found no significant relationship between them. In other words, when students are taught to save environment and pursue sustainability and harmony for future generations, their sustainability-related values (which tend towards materialism) may change, leading them to adopt pro-environmental attitudes that support sustainability and counter the effects of GCC.

**Figure 2:**
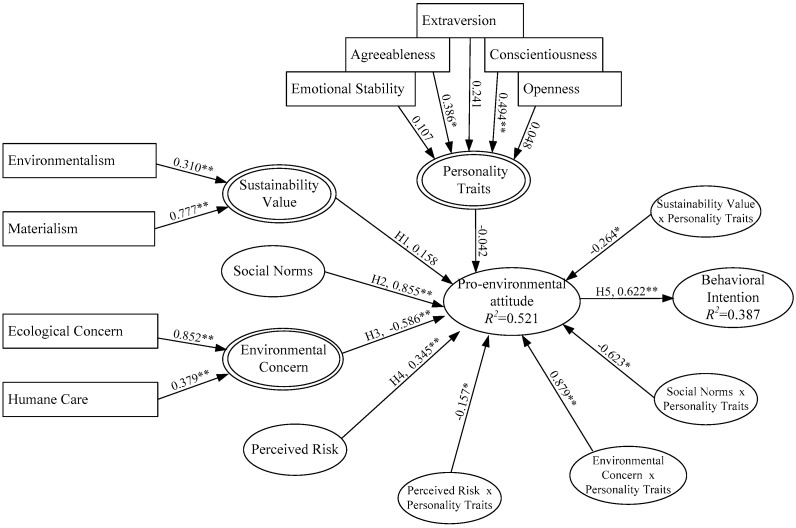
Path relations of the proposed model (including interaction effects). * *p* < 0.05; ** *p* < 0.01.

We used the PLS method to estimate the important path relationships among the dimensions (see Figure 2), with standardized coefficients adopted for each path value. Among the six main effect path relationships, four assumptions reached significance (α = 0.05): social norms on pro-environmental attitude (H2, 0.885), environmental concern on pro-environmental attitude (H3, −0.586), perceived risk on pro-environmental attitude (H4, 0.345) and pro-environmental attitude on behavioral intention (H5, 0.622). The correlation (H1) between sustainability value and pro-environmental attitude achieved insignificance. Mass media is the primary outlet for GCC issues and the connection between media broadcasts and emotional impressions could encourage individuals through social pressure to adopt pro-environmental attitudes and to increase their awareness of GCC. The social norm on pro-environmental attitude path (0.855) indicates that a significant relationship existed between social norms and pro-environmental attitudes. In terms of the perceived risk, perceived risk on pro-environmental attitude path (0.345) associated higher perceived risks of GCC with stronger expressions of pro-environmental attitudes. The pro-environmental attitude on behavioral intention path (0.622) indicates that pro-environmental attitude had a significant and positive impact on behavioral intention. These findings are consistent with the empirical results in Milfont [5], which showed that an individual’s commitment to lessening the impact of GCC reflects positive attitudes. 

With regard to the explanatory power of the connections among perceived risks, beliefs and attitudes, the present study found pro-environmental attitudes were significantly and positively affected by perceived risk and social norms but significantly and negatively by environmental concern. No significant effect was noted for sustainability value. The explanatory power of variation (*R^2^*) for pro-environmental attitudes was 0.521. However, the results empirically support the chain connection between pro-environmental attitudes and behavioral intention, which is consistent with the literatures [17,75], which generally emphasizes this strong connection. The structural model explained 38.7% of the variance in participant responses regarding intentions to adopt the pro-environmental behavior. Laudenslager et al. [75] reported that the core constructs of the TPB accounted for variance in intent ranging from 21% to 35% across three pro-environment behavioral intentions.

### 4.4. The Impact of Personality Traits on the Response to GCC

Related studies [76,77] have shown that an individual’s perspective may be mostly described with five personality traits of agreeableness, extraversion, openness, conscientiousness and emotional stability. To highlight explanation degrees and abstraction of latent variables (personality traits), the present study used confirmatory factor analysis to verify stability of personality traits to measure pro-environment attitude. Personality traits were set as the formative indicators to test the stability of predictive variables. Only agreeableness and conscientiousness achieved statistical significance. Conscientiousness had the highest associations with environmental personal traits and the lowest associations for openness.

Following Costa and McCrea [54] and Goldberg [78], the present study used factor analysis to categorize measurement variables for personality traits into five latent variables, with the explicit behaviors of an individual constituting these variables. Fraj and Martinez [79] and Moody and Hartel [53] demonstrated that a high sense of environmental responsibility increased the likelihood of individuals purchasing environmentally friendly products. Table 3 shows the results for moderator hypotheses (H6 to H9). Adding the interaction terms increased the explained variance at step 3 significantly. Integrating the effects of other interactions, no evidence was found that personality traits influence the pro-environmental attitudes (−0.042). The analytical results reported four moderation effects for the relationship between predictors (sustainability value, social norms, environmental concern and perceived risk) and pro-environmental attitude, which supported all of the hypotheses.

## 5. Discussion

Understanding the complex nature of general behavioral tendencies is necessary in order to maximize changes in specific behavior-related intentions and attitudes. The present study introduced a solid green-education-based theoretical model [10,80] to predict the pro-environment behavioral intention of undergraduate students based on VBN and TPB models to address undergraduate students’ responses to GCC adaptations strategies. Green education is one of the major adaptation strategies used to encourage a positive public response to GCC [80]. The behavioral chains of the proposed model included sustainability values, environmental beliefs and social norms and personality traits were the main moderator. The model provides an effective prediction of pro-environment behavioral intention based on the value–attitude–behavior chain that students followed when dealing with GCC issues.

### 5.1. Implications of the Proposed Model for Values, Beliefs and Norms

According to relevant theories of environmental behaviors, values, beliefs and norms, are potentially promoting factors to influence pro-environmental behaviors; all are positively related to pro-environmental attitudes. In the value-attitude-behavior chain process, environmentalism and materialism are the antecedent variables for sustainability values. Ecological concern and humane care are the constituent aspects of environmental concern. While several researchers [81,82,83] used simulations or constructive substitution variables as indicators for environmental attitudes, the present investigation interpreted ecological concern and humane care as distinct issues.

In terms of sustainability value, people are deemed to realize the importance of sustainability value when they become aware of root causes of GCC [27,38,57]. At this point, people are encouraged to raise their level of environmental concern, adopt criteria for their behavioral beliefs and maintain a positive relationship among values, beliefs and pro-environment intentions. Due to positive effect that environmental concern has on environmental attitudes [21,39], environmental concern and pro-environmental attitude have strong resonance with one another. Notably, a direct and significant relationship exists between sustainability value and environmentalism. However, when individuals believe that their actions in response to environmental concerns are unable to ameliorate the effects of GCC, they review their sustainability values and time commitments in light of their position on the spectrum between environmentalism and materialism. Because the effects of GCC adaptation strategies take a long time to manifest and are not immediately apparent, the pro-environmental attitudes of an individual lack positive and visible feedback/incentives, which weakens individual environmental attitudes with regard to improving GCC.

The present study categorized GCC beliefs into pro-environmental attitudes and behavioral intentions. Stern [21] advocated that values induce changes in behavior and affect the environmental beliefs of individuals. In the present study, materialism (0.777) and environmentalism (0.310) significantly affected sustainability value. Therefore, for the participants, sustainability values were mainly the results of materialism and the economic and practical benefits of materialism were higher than those of environmentalism. Participants commonly indicated that they held involuntary self-care for the environment, making it unlikely that the mainstream consciousness of environmentalism (e.g., carbon-reduction and a focus on environmental issues) inspires undergraduate students to adopt a pro-environmental response/action.

Focusing on the factors that affect beliefs and norms, Bamberg and Moser [81] argued that an individual’s social norms are also a type of belief. Therefore, this belief may be integrated into social norms as a predictor of attitudes, beliefs, or behavioral intentions [4,21,84]. The present study incorporated the references of individuals to society and social groups as measurement questions and did not use the operational definitions of self-identity proposed by Mannetti et al. [85] and White and Hyde [86]. Social pressure is likely a factor that encourages powerful individuals to engage in consistent pro-environmental behavior and to pressure others to follow this behavior. This reflects the pressure that the social collective consciousness exerts on personal moral responsibility and sentiment [38]. Roeser [87] and Shackelford [88] contend that the norms of social groups have significant and permanent effects on the boundaries of pro-environmental behaviors and attitudes. The results of the present study are consistent with these previous studies.

The behavior of laypeople differs from scientists who observe and are in contact with data about global warming on a daily basis. The journal Science reviewed 928 articles on the topic of GCC, with all of the articles concluding that there exists a significant causal relationship between human activities and GCC. Oreskes [89] pointed to the dangers of GCC, showing that global warming has become more evident. These GCC risk messages, also communicated through and edited by scientists and media organizations via mass media, warn of the dangers by focusing on interactions among the scientific evidence. The results of the present study indicate that undergraduate students develop a higher awareness of the risks of GCC after becoming more aware of the potential related dangers, which is a process that both invokes these students’ sentimental values and changes their impressions of GCC [33,36]. The creation of these important psychological links often results in students more actively volunteering to perform amelioration activities and indicating their preferences for public policies that are intended to reduce carbon emissions while positively affecting pro-environmental attitudes.

Finally, for those people wanting to adopt environment-friendly measures, they must have sufficient motivation and willingness [90]. It may be reasonable to believe that people who maintain a high level of personal interest also maintain their behaviors until they satisfy self-oriented motives and attitudes [91,92]. However, the results of the present study show the path relationship between pro-environmental attitude and behavioral intention to be significant, which is consistent with the inferred results of previous models for environmental behavior [5,16,38,93]. Exposing people to the scientific consensus on climate change is inadequate. The resultant attitudes toward GCC must translate into positive actions such as green consumerism, reduction of carbon emissions and recycling. According to the results of the present study, when undergraduate students adopt an ethic of environmental concern, they abandon past misconceptions, such as the idea that the government and manufacturers are solely responsible to resolve GCC issues. Furthermore, they express greater concern for ecology, support public policies that improve GCC, adopt behavioral intentions to change past consumer behaviors, believe that sustainability values and weaken the influence of materialism and develop a deeper sense of environmental concern and a greater understanding of the perceived risks of GCC.

### 5.2. The Effects of Personality Traits on Values, Beliefs and Norms

Examining the relationship between the dimensions of Big Five model and GCC related issues bridges connections with the environment and personality on GCC adaptation strategies. A clear and negative relationship was found between the interaction effects of personality traits and sustainability value on the one hand and of personality traits and environmental attitudes on the other. The combination of high levels in particular traits (i.e., strong conscientiousness and agreeableness) with low-scoring sustainability value (i.e., weak materialism and environmentalism) resulted in a negative relationship with pro-environmental attitudes. These results indicate a pro-environmental attitude is not sufficient to improve sustainability value. Alternatively, the combination of low-scoring personality traits with high-scoring sustainability value (i.e., strong materialism and environmentalism) resulted in a positive relationship with pro-environmental attitude. In other words, individuals with low-scoring sustainability value are unlikely to show any improvement in pro-environmental attitudes despite their high levels in particular traits. Only those with high-scoring sustainability value may be expected to develop better and more positive pro-environmental attitudes. We theorize that pro-environmental attitude is both linked to antecedent and moderator variables, the strongest links being conscientiousness and agreeableness. This might explain the link between pro-environmental attitude on GCC issues and the need to incorporate unexpected environmental outcomes in daily life.

After integrating the moderating effects of personality traits, the relationship of environmental concern influenced pro-environment attitudes (−0.586), indicating that the more one cares about ecology and humanity, the fewer preferences (likes or dislikes) one has pro-environmental attitudes. For individuals with low-scoring personality traits, the correlation coefficient of environmental concern on the pro-environmental attitudes was negative, while the scores for pro-environmental attitudes increased steadily with higher scores for environmental concern. In contrast, individuals with high levels of particular traits, regardless of their level of environmental concern, showed better pro-environmental attitudes than their low-scoring peers. This result demonstrates a conflict with the results of Guagnano et al. [94] and López-Mosquera and Sánchez [47]. Because an individual’s psychology is the starting point for environmental concerns, personality traits may positively moderate the relationship between environmental concern and attitudes.

When considering personality traits and social norms, the present study found that positive social norms strengthen pro-environmental attitudes with high levels in particular traits. Conversely, individuals with low-scoring personality traits experience poorer social norms, which negatively affect their pro-environmental attitudes. However, pro-environmental attitudes improved rapidly with increased social norms along a track that was similar to peers with high levels in particular traits. Changes to environmental behaviors are an ongoing activity. All of the following may influence an individual’s personal norms: improvements to sustainable concerns and environmental concerns (e.g., incentives and rewards, product distribution process and raw material transformation), changes in personal beliefs and pro-environmental attitudes (e.g., carbon-reduction habits and friendliness toward the environment), support and commitment from important figures and social norms. This is especially true when influential individuals or groups participate in activities that are designed to help prevent GCC, which introduces a positive image and an improved quality of life.

Boons, et al. [27] and Owens [95] posited that distributing appropriate information helps improve popular perceived risks and increase environmental concern over GCC among uninformed people. When people aware of the environmental risks of GCC, the relevant scientific principles and technologies that are easy to understand [36,96,97], which can be used to develop concepts for reducing environmental risks. Preventive measures should also be taken to protect the future and meaningful operational strategies should be established. The more individuals with high- and low-scoring personality traits understand the environmental risks of GCC, the stronger their pro-environmental attitudes should become. For both groups of individuals, changes in their pro-environmental attitudes and their understanding of perceived risks of GCC tend to be consistent. An important step in the theoretical development of this approach is to examine the relationships among currently available instruments and variables and how they combine to predict environmental behavior. The present study’s premise was that individuals engage in pro-environment behavioral intentions of GCC adaptation to the degree that is allowed by their pro-environmental attitudes. The results show that the theoretical and methodological background of green educators can be enriched by using antecedent variables for pro-environment behavioral intentions within survey procedures. This is a powerful predictive model of self-reported pro-environment behavioral intention as well as provides a better understanding of the structure of individual personality traits.

### 5.3. Research Limitations

Common method bias is often observed with self-reported questionnaires. In addition to alternating the application of various questionnaire design methods (including respondents’ confidentiality, the confidentiality of the meaning of questions and reversed questions), the present study placed behavior-oriented measurement questions at the end of the questionnaire. Analyzing the covariance matrices in order to verify the post hoc model thus reduced the potential impact of covariance.

## 6. Conclusions

In Asia, there is a growing consensus that individuals need to promote sustainable consumption and build a sustainable society. The current empirical study extended the literature by identifying perceived risk, personality traits, VBN theory and TPB model and proposed a moderating model for pro-environmental behavioral intention of undergraduate students with structural equation modeling. This model predicts relationships between factors of pro-environmental behavioral intention, environmental concern, sustainability value, social norms, pro-environmental attitude and perceived risk of climate change. It also provides improvements to the measurement of personality trait mitigation by considering roles of sustainability value, environmental concern, social norms, perceived risk and pro-environmental attitude. The findings also highlight the psychological significance of personality traits and antecedent constructs pro-environmental attitude to climate change. The effects of personality on the antecedents of pro-environment behavioral intention cannot be overstated; the current findings are strong encouragement for future research in this field. Overall, our study demonstrates that personal traits may pose some barriers to encourage pro-environmental attitude for undergraduate students and could be served as a useful framework for further investigations. Green education could improve pro-environmental attitude and build its connections to the planet, value and concern for undergraduate students and they may behave more eco-friendly manners. From a practical perspective, understanding of driving or deterring factors of the pro-environmental attitude and behavior intention can better inform green educators and sustainability policy managers in Taiwan. By expanding the scope of research beyond traditional pro-environment behavioral intentions, this conceptual model helps clarify how pro-environmental behaviors can help fight climate change and it is a call for further research. Further research is necessary to explain the detailed interactions of personal traits and pro-environmental behavior related to GCC issues—for example, actors of participants, situational activators and information sources of participants [98,99,100].

## Figures and Tables

**Table 1:** Statistics of research participant.

Variable	Type	Number	Percentage (%)
Gender	Male	90	32.7
	Female	185	67.3
Grade	First year	39	14.2
	Second year	128	46.5
	Third year	65	23.6
	Fourth year	37	13.5
	Missing value	6	2.2
Time of first awareness of GCC	Less than a year ago	20	7.3
	1–2 years ago	39	14.2
	3–4 years ago	104	37.8
	5–6 years ago	73	26.5
	7 or more years ago	36	13.1
	Missing value	3	1.1
Average age		21.1	

GCC: global climate change.

**Table 2:** Reliability and validity indicators of the proposed model.

Items	AVE	Composite Reliability	*R^2^*
Pro-environmental Attitude	0.771	0.931	0.521
Behavioral Intention	0.652	0.882	0.387
Environmental Concern	0.510	0.789	-
Sustainability Value	0.568	0.884	-
Ecological Concern	0.910	0.953	-
Humane Care	0.807	0.893	-
Environmentalism	0.647	0.783	-
Materialism	0.690	0.899	-
Social Norms	0.910	0.968	-
Perceived Risk	0.814	0.929	-
Environmental Concern × Personality Traits	0.485	0.946	-
Sustainability Value × Personality Traits	0.452	0.960	-
Social Norms × Personality Traits	0.779	0.981	-
Perceived Risk × Personality Traits	0.452	0.905	-

AVE: Average Variance Extracted.

**Table 3:** Path coefficients of the research model.

Path Coefficients/Models	Model 1	Model 2	Model 3
Sustainability value → Pro-environmental attitude	−0.092	−0.092	0.158
Social norms → Pro-environmental attitude	0.436 **	0.402 **	0.855 **
Environmental concern → Pro-environmental attitude	0.061	0.036	−0.586 **
Perceived risk → Pro-environmental attitude	0.360 **	0.339 **	0.345 **
Pro-environmental attitude → Behavioral intention	0.622 **	0.622 **	0.622 **
Moderator main effect			
Personality traits → Pro-environmental attitude	-	0.121 *	−0.042
Moderator interaction effect			
Personality traits × Sustainability value → Pro-environmental attitude	-	-	−0.264 *
Personality traits × Social norms → Pro-environmental attitude	-	-	−0.623 *
Personality traits × Environmental concern → Pro-environmental attitude	-	-	0.879 **
Personality traits × Perceived risk → Pro-environmental attitude	-	-	−0.157 *
Pro-environmental attitude *R^2^*	0.487	0.498	0.521
Pro-environmental attitude △*R^2^*	-	0.011	0.023
Behavioral intention *R^2^*	0.387	0.387	0.387

* *p* < 0.05; ** *p* < 0.01.

## Appendix A

**Table A1:** Questions assessing perceived risk and pro-environmental behavioral intention.

Construct/Sub-Construct		Question
Personality traits	Extraversion	I see myself as enthusiastic.
		I see myself as quiet. (R)
	Agreeableness	I see myself as warm.
		I see myself as quarrelsome. (R)
	Conscientiousness	I see myself as self-disciplined.
		I see myself as careless. (R)
	Emotional stability	I see myself as easily upset.
		I see myself as emotionally stable. (R)
	Openness	I see myself as open to new experiences.
		I see myself as uncreative. (R)
Sustainability value	Environmentalism	The balance of nature is very delicate and easily upset.
		Humans were meant to rule over the rest of nature. (R)
		We are approaching the limit to the number of people the earth can support.
	Materialism	We respect people who choose to live according to their own moral standards.
		The earth has plenty of natural resources if we just learn how to develop them. (R)
		If humans are severely abusing the environment, we will soon experience a major ecological catastrophe. (R)
Environmental concern	Ecological concern	Climate change is a serious threat to the welfare of future generations and environment.
		Rising global temperatures due to climate change are serious.
		The impact of climate change on the rivers and coasts of Taiwan are serious.
	Humane care	Humans have the right to modify the natural environment to suit their needs. (R)
		Animals and plants have the same right as humans to exist.
		Humans are severely abusing the environment.
Social norms	My classmates expect me to reduce CO_2_ emissions.	
	Faculty and staff in schools expect me to reduce CO_2_ emissions.	
	My family members expect me to reduce CO_2_ emissions.	
	My friends expect me to reduce CO_2_ emissions.	
Perceived risk	Climate change is an acceptable risk. (R)	
	The effects of climate change are likely to be catastrophic.	
	Recent typhoons and extreme temperature events in Taiwan are the result of climate change.	
	Climate change damages the natural environment and wildlife in Taiwan.	
Environmental attitude	Reducing CO_2_ emissions is a good idea.	
	Reducing CO_2_ emissions would be beneficial to me.	
	Reducing CO_2_ emissions would be enjoyable.	
	Reducing CO_2_ emissions would be my favorite activity.	
Behavioral intention	Use cars less; buy more efficient products.	
	I am willing to change my lifestyle to counteract climate change.	
	Become “carbon neutral” using offsets as necessary.	
	Make major efforts to adapt climate change.	
	Do everything that can reduce the impact of climate change.	

R: denotes reverse scored items.

## References

[1] IPCC AR4 WG1 (2007). Climate Change 2007: The Physical Science Basis, Contribution of Working Group I to the Fourth Assessment Report of the Intergovernmental Panel on Climate Change.

[2] World Health Organisation (2008). Protecting Health from Climate Change—A Toolkit for Event Organisers. http://www.who.int/world-health-day/toolkit/toolkit_en.pdf.

[3] Kellstedt P.M., Zahran S., Vedlitz A. (2008). Personal efficacy, the information environment, and attitudes toward global warming and climate change in the United States. Risk Anal..

[4] Lin S.P. (2015). Raising Public Awareness: The role of the household sector in mitigating climate change. Int. J. Environ. Res. Public Health.

[5] Milfont T.L. (2012). The interplay between knowledge, perceived efficacy, and concern about global warming and climate change: A one-year longitudinal study. Risk Anal..

[6] O’Connor R.E., Bord R.J., Fisher A. (1999). Risk perceptions, general environmental beliefs, and willingness to address climate change. Risk Anal..

[7] Stedman R.C. (2004). Risk and Climate Change: Perceptions of key policy actors in Canada. Risk Anal..

[8] Wright T.S., Wilton H. (2012). Facilities management directors’ conceptualizations of sustainability in higher education. J. Clean. Prod..

[9] Barr S. (2007). Factors influencing environmental attitudes and behaviors: A UK case study of household waste management. Environ. Behav..

[10] Horhota M., Asman J., Stratton J.P., Halfacre A.C. (2014). Identifying behavioral barriers to campus sustainability: A multi-method approach. Int. J. Sust. Higher Ed..

[11] Wachholz S., Artz N., Chene D. (2014). Warming to the idea: University students’ knowledge and attitudes about climate change. Int. J. Sust. Higher Ed..

[12] Erdogan M., Akbunar S., Asik U.O., Kaplan H., Kayir C.G. (2012). The effects of demographic variables on students’ responsible environmental behaviors. Procedia Soc. Behav. Sci..

[13] Zsóka Á., Szerényi Z.M., Széchy A., Kocsis T. (2013). Greening due to environmental education? Environmental knowledge, attitudes, consumer behavior and everyday pro-environmental activities of Hungarian high school and university students. J. Clean. Prod..

[14] Hungerford H.R., Volk T.L. (1990). Changing learner behavior through environmental education. J. Environ. Educ..

[15] Ajzen I. (1991). The theory of planned behavior. Organ. Behav. Hum. Decis. Process..

[16] Fielding K.S., McDonald R., Louis W.R. (2008). Theory of planned behaviour, identity and intentions to engage in environmental action. J. Environ. Psychol..

[17] Greaves M., Zibarras L.D., Stride C. (2013). Using the theory of planned behavior to explore environmental behavioral intentions in the workplace. J. Environ. Psychol..

[18] Shove E. (2010). Beyond the ABC: Climate change policy and theories of social change. Environ. Plan. A.

[19] Ajzen I. (2011). The theory of planned behaviour: Reactions and reflections. Psychol. Health.

[20] Mancha R.M., Yoder C.Y. (2015). Cultural antecedents of green behavioral intent: An environmental theory of planned behavior. J. Environ. Psychol..

[21] Stern P.C. (2000). Toward a coherent theory of environmentally significant behavior. J. Soc. Issues.

[22] Schwartz S.H. (1994). Are there universal aspects in the structure and contents of human values?. J. Soc. Issues.

[23] Oreg S., Katz-Gerro T. (2006). Predicting proenvironmental behavior cross-nationally values, the theory of planned behavior, and value-belief-norm theory. Environ. Behav..

[24] Steg L., Bolderdijk J.W., Keizer K., Perlaviciute G. (2014). An integrated framework for encouraging pro-environmental behaviour: The role of values, situational factors and goals. J. Environ. Psychol..

[25] Steg L., Dreijerink L., Abrahamse W. (2005). Factors influencing the acceptability of energy policies: A test of VBN theory. J. Environ. Psychol..

[26] Taylor S., Todd P. (1997). Understanding the determinants of consumer composting behavior. J. Appl. Soc. Psychol..

[27] Boons F., Lüdeke-Freund F. (2013). Business models for sustainable innovation: State-of-the-art and steps towards a research agenda. J. Clean. Prod..

[28] Dunlap R.E., Van Liere K.D., Mertig A.G., Jones R.E. (2000). New trends in measuring environmental attitudes: Measuring endorsement of the new ecological paradigm: A revised NEP scale. J. Soc. Issues.

[29] Lin P.C., Huang Y.H. (2012). The influence factors on choice behavior regarding green products based on the theory of consumption values. J. Clean. Prod..

[30] Van Riper C.J., Kyle G.T. (2014). Understanding the internal processes of behavioral engagement in a national park: A latent variable path analysis of the value-belief-norm theory. J. Environ. Psychol..

[31] Hurst M., Dittmar H., Bond R., Kasser T. (2013). The relationship between materialistic values and environmental attitudes and behaviors: A meta-analysis. J. Environ. Psychol..

[32] Liobikienė G., Juknys R. (2016). The role of values, environmental risk perception, awareness of consequences, and willingness to assume responsibility for environmentally-friendly behaviour: The Lithuanian case. J. Clean. Prod..

[33] Leiserowitz A. (2006). Climate change risk perception and policy preferences: The role of affect, imagery and values. Clim. Chang..

[34] Steurer R., Langer M.E., Konrad A., Martinuzzi A. (2005). Corporation stakeholders and sustainable development I: A theoretical exploration of business society relations. J. Bus. Ethics.

[35] Ferdig M.A. (2007). Sustainability leadership: Co-creating a sustainable future. J. Chang. Manag..

[36] Akerlof K., Rowan K.E., Fitzgerald D., Cedeno A.Y. (2012). Communication of climate projections in U.S. media amid politicization of model science. Nat. Clim. Chang..

[37] Brody S.D., Zahran S., Vedlitz A., Grover H. (2008). Examining the relationship between physical vulnerability and public perceptions of global climate change in the United States. Environ. Behav..

[38] Fusco E., Snider A., Luo S. (2012). Perception of global climate change as a mediator of the effects of major and religious affiliation on college students’ environmentally responsible behavior. Environ. Educ. Res..

[39] Leiserowitz A.A., Kates R.W., Parris T.M. (2006). Sustainability values, attitudes, and behaviors: A review of multinational and global trends. Annu. Rev. Environ. Res..

[40] Giddens A. (2009). The Politics of Climate Change.

[41] Bostrom A., Morgan M.G., Fischhoff B., Read D. (1994). What do people know about global climate change?. Risk Anal..

[42] Read D., Bostrum A., Morgan M.G., Fischoff B., Smuts T. (1994). What do people know about global climate change?. Risk Anal..

[43] Shephard K., Harraway J., Lovelock B., Skeaff S., Slooten L., Strack M., Furmari M., Jowett T. (2014). Is the environmental literacy of university students measureable?. Environ. Educ. Res..

[44] Boykoff M., Boykoff J. (2004). Bias as balance: Global warming and the U.S. prestige press. Glob. Environ. Chang..

[45] Bord R.J., O’Connor R.E., Fisher A.A. (2000). In what sense does the public need to understand global climate change?. Public Underst. Sci..

[46] Hutchinson J., Prady S.L., Smith M.A., White P.C., Graham H.M. (2015). A scoping review of observational studies examining relationships between environmental behaviors and health behaviors. Int. J. Environ. Res. Public Health.

[47] López-Mosquera N., Sánchez M. (2012). Theory of planned behavior and the value-belief-norm theory explaining willingness to pay for a suburban park. J. Environ. Manag..

[48] Kaiser F.G., Hubner G., Bogner F.X. (2005). Contrasting the theory of planned behavior with the value-belief-norm model in explaining conservation behaviour. J. Appl. Soc. Psychol..

[49] Erdogan M., Ok A., Marcinkowski T.J. (2012). Development and validation of children’s responsible environmental behavior scale. Environ. Educ. Res..

[50] Ojedokun O. (2011). Attitude towards littering as a mediator of the relationship between personality attributes and responsible environmental behavior. Waste Manag..

[51] Simmons D., Widmar R. (1990). Motivations and barriers to recycling: Toward a strategy for public education. J. Environ. Educ..

[52] Hovardas T., Poirazidis K. (2006). Evaluation of the environmentalist dimension of ecotourism at the Dadia Forest Reserve (Greece). Environ. Manag..

[53] Moody G.L., Hartel P.G. (2007). Evaluating an environmental literacy requirement chosen as a method to produce environmentally literate university students. Int. J. Sustain. High. Educ..

[54] Costa P.T., McCrac R.R. (1992). Revised NEO Personality Inventory (NEO-PI-R) and NEO Five-Factor Inventory (NEO-FFI), Professional Manual.

[55] Brick C., Lewis G.J. (2016). Unearthing the “green” personality: Core traits predict environmentally friendly behavior. Environ. Behav..

[56] Wood S. (2012). Prone to progress: Using personality to identify supporters of innovative social entrepreneurship. J. Public Policy Mark..

[57] Milfont T.L., Sibley C.G. (2012). The big five personality traits and environmental engagement: Associations at the individual and societal level. J. Environ. Psychol..

[58] Hirsh J.B. (2010). Personality and environmental concern. J. Environ. Psychol..

[59] Ringle C.M., Wende S., Will A. (2005). SmartPLS—Version 2.0.

[60] Chin W.W. (1998). Issues and opinion on structural equation modeling. MIS Quart..

[61] Petter S., Straub D., Rai A. (2007). Specifying formative constructs in information systems research. MIS Quart..

[62] Cohen J., Cohen P., West S.G., Aiken L.S. (2013). Applied Multiple Regression/Correlation Analysis for the Behavioral Sciences.

[63] Marsh H.W., Wen Z., Hau K.T. (2004). Structural equation models of latent interactions: Evaluation of alternative estimation strategies and indicator construction. Psychol. Methods.

[64] Gifford R. (2008). Psychology’s essential role in alleviating the impacts of climate change. Can. Psychol..

[65] Hair F., Black W.C., Babin B.J., Anderson R.E. (2010). Multivariate Data Analysis: A Global Perspective.

[66] Anderson J.C., Gerbing D.W. (1988). Structural equation modeling in practice: A review and recommended two-step approach. Psychol. Bull..

[67] Fornell C., Larcker D.F. (1981). Evaluating structural equation models with unobservable variables and measurement error. J. Mark. Res..

[68] Bagozzi R.P., Yi Y. (1988). On the evaluation of structural equation models. J. Acad. Mark. Sci..

[69] Tenenhaus M., Vinzi V.E., Chatelin Y.M., Lauro C. (2005). PLS path modeling. Comput. Stat. Data Anal..

[70] Marcoulides G.A., Chin W.W., Saunders C. (2009). A critical look at partial least squares modeling. MIS Quart..

[71] Chin W.W., Marcolin B.L., Newsted P.R. (2003). A partial least squares latent variable modeling approach for measuring interaction effects: Results from a Monte Carlo simulation study and voice mail emotion/adoption study. Inf. Syst. Res..

[72] Baron R.M., Kenny D.A. (1986). The moderator-mediator variable distinction in social psychological research: Conceptual, strategic, and statistical considerations. J. Pers. Soc. Psychol..

[73] Kenny D.A. (2008). Reflections on mediation. Organ. Res. Method.

[74] De Groot J.I., Steg L. (2008). Value orientations to explain beliefs related to environmental significant behavior: How to measure egoistic, altruistic, and biospheric value orientations. Environ. Behav..

[75] Laudenslager M.S., Holt D.T., Lofgren S.T. (2004). Understanding air force members’ intentions to participate in pro-environmental behaviors: An application of the theory of planned behavior. Percept. Mot. Skills.

[76] Barrick M.R., Mount M.K. (1991). The big five personality dimensions and job performance: A meta-analysis. Pers. Psychol..

[77] Judge T.A., Higgins C.A., Thoresen C.J., Barrick M.R. (1999). The big five personality traits, general mental ability, and career success across the life span. Pers. Psychol..

[78] Goldberg L.R. (1990). An alternative “description of personality”: The big-five factor structure. J. Pers. Soc. Psychol..

[79] Fraj E., Martinez E. (2006). Environmental values and lifestyles as determining factors of ecological consumer behaviour: An emprical analysis. J. Consum. Mark..

[80] Eagle L., Low D., Case P., Vandommele L. (2015). Attitudes of undergraduate business students toward sustainability issues. Int. J. Sust. Higher Ed..

[81] Bamberg S., Möser G. (2007). Twenty years after Hines, Hungerford, and Tomera: A new meta-analysis of psychosocial determinants of pro-environmental behavior. J. Environ. Psychol..

[82] Homburg A., Stolberg A. (2006). Explaining pro-environmental behavior with a cognitive theory of stress. J. Environ. Psychol..

[83] Meinhold J.I., Malkus A.J. (2005). Adolescent environmental behaviors. Can knowledge, attitudes, and self-efficacy make a difference?. Environ. Behav..

[84] Shephard K., Harraway J., Jowett T., Lovelock B., Skeaff S., Slooten L., Strack M., Furmari M. (2015). Longitudinal analysis of the environmental attitudes of university students. Environ. Educ. Res..

[85] Mannetti L., Pierro A., Livi S. (2004). Recycling: Planned and self-expressive behaviour. J. Environ. Psychol..

[86] White K.M., Hyde M.K. (2012). The role of self-perceptions in the prediction of household recycling behavior in Australia. Environ. Behav..

[87] Roeser S. (2012). Risk communication, public engagement, and climate change. Risk Anal..

[88] Shackelford T.K. (2006). Recycling, evolution and the structure of human personality. Pers. Individ. Differ..

[89] Oreskes N. (2004). The scientific consensus on climate change. Science.

[90] Kotchen M.J., Reiling S.D. (2000). Environmental attitudes, motivations, and contingent valuation of nonuse values: A case study involving endangered species. Ecol. Econ..

[91] Pelletier L.G. (2002). A motivational analysis of self-determination for pro-environmental behaviors. Handbook of Self-Determination Research.

[92] De Groot J.I., Steg L. (2010). Relationships between value orientations, self-determined motivational types and pro-environmental behavioural intentions. J. Environ. Psychol..

[93] Nigbur D., Lyons E., Uzzell D. (2010). Attitudes, norms, identity and environmental behaviour: Using an expanded theory of planned behaviour to predict participation in a kerbside recycling programme. Br. J. Soc. Psychol..

[94] Guagnano G.A., Stern P.C., Dietz T. (1995). Influences on attitude-behavior relationships. A natural experiment with curbside recycling. Environ. Behav..

[95] Owens S. (2000). Engaging the public: Information and deliberation in environmental policy. Environ. Plan. A.

[96] Slovic P., Finucane M.L., Peters E., MacGregor D.G. (2004). Risk as analysis and risk as feelings: Some thoughts about affect, reason, risk, and rationality. Risk Anal..

[97] Weber E.U., Stern P.C. (2011). Public understanding of climate change in the United States. Am. Psychol..

[98] Kallgren C.A., Wood W. (1986). Access to attitude-relevant information in memory as a determinant of attitude-behavior consistency. J. Exp. Soc. Psychol..

[99] Harland P., Staats H., Wilke H.A. (2007). Situational and personality factors as direct or personal norm mediated predictors of pro-environmental behavior: Questions derived from norm-activation theory. Basic Appl. Soc. Psychol..

[100] Soliño M., Farizo B.A. (2014). Personal traits underlying environmental preferences: A discrete choice experiment. PLoS ONE.

